# Angiostatic actions of capsicodendrin through selective inhibition of VEGFR2-mediated AKT signaling and disregulated autophagy

**DOI:** 10.18632/oncotarget.9307

**Published:** 2016-05-11

**Authors:** Christopher C. Pan, Nirav Shah, Sanjay Kumar, Sarah E. Wheeler, Jason Cinti, Dale G. Hoyt, Christine E. Beattie, Min An, Karthikeyan Mythreye, L. Harinantenaina Rakotondraibe, Nam Y. Lee

**Affiliations:** ^1^ Division of Pharmacology, College of Pharmacy, The Ohio State University, OH, USA; ^2^ Department of Chemistry and Biochemistry, University of South Carolina, Columbia, SC, USA; ^3^ Division of Medicinal Chemistry and Pharmacognosy, College of Pharmacy, The Ohio State University, OH, USA; ^4^ Davis Heart Lung Research Institute, The Ohio State University, OH, USA; ^5^ Comprehensive Cancer Center, The Ohio State University, OH, USA; ^6^ Department of Neuroscience, The Ohio State University, OH, USA

**Keywords:** autophagy, AKT, VEGF, angiogenesis, endothelium

## Abstract

Angiogenesis is the formation of new blood vessels from existing vasculature critical for embryonic development and vascular remodeling. Its dysregulation underlies numerous pathologic states ranging from ischemia to tumor growth and as such identifying new targeted- therapies is of significant interest for angiogenesis-based medicine. Here we evaluated the potential angiostatic properties of capsicodendrin (CPCD), a natural compound isolated from *Cinnamosma macrocarpa*, a plant belonging to the Malagasy Cinnamosma. CPCD potently inhibits endothelial proliferation, migration and capillary tube formation at nanomolar to low micromolar concentrations without inducing cytotoxic effects. We show that CPCD directly inactivates VEGFR2 and downstream AKT signaling, thereby strongly inducing autophagy as determined by increased expression of beclin1, autophagy-related gene (Atg) 3, Atg5 and LC3 cleavage. Ectopic AKT overexpression counteracts the inhibitory effects of CPCD on proliferation and capillary tubule formation. Importantly, CPCD treatment *in vivo* inhibits sprouting angiogenesis as evidenced by strongly reduced intersegmental vessel (ISV) sprouting and subintestinal vessel (SIV) formation during zebrafish embryonic development, and correlates with increased presence of LC3II along the ISVs despite overall reduced vasculature. These findings demonstrate CPCD as a potent inhibitor of the VEGFR2/AKT pathway at nanomolar concentrations and inducer of autophagy-related angiostatic effects.

## INTRODUCTION

Angiogenesis is the formation of new blood vessels from pre-existing vasculature, a highly coordinated multi-step process involving endothelial matrix degradation, migration, proliferation and capillary tube formation [1]. Numerous therapeutic strategies are aimed at revascularizing ischemic or damaged tissues, and conversely, inhibiting angiogenesis in tumors and proliferative retinopathies [2–4]. While many anti-angiogenic treatments demonstrate limited long-term efficacy due to evasive or intrinsic resistance, improvements on progression-free survival among patients with various degrees of metastatic and advanced cancers are still achieved when combined with conventional chemotherapy, and anti-angiogenic therapies may have clear benefits in normalizing tumor vessels for better drug delivery resistance [5–7].

A number of new classes of therapeutic agents are being developed, and natural products represent an important source of novel drug leads and the basis for many rational synthetic designs [8–12]. A growing number of small molecules have been identified as promising agents with dozens in preclinical and clinical trials including the flavonoids. These and other natural products and metabolites act through multiple interdependent mechanisms including apoptosis, matrix metalloproteinase-2 (MMP2) downregulation, and quite often, by suppressing vascular endothelial growth factor (VEGF) gene expression [13]. Interestingly, while their multitude of inhibitory targets could certainly work in favor of reducing the development of resistance by cancer cells, concerns of unpredictable or undesirable off-target effects remain a significant challenge in clinical settings.

As the plant species in the Canellaceae family endemic to Madagascar, three species- *Cinnamosma fragrans, C. macrocarpa* and *C. madagascariensis* are all widely used to alleviate a range of symptoms including malaria and fatigue, and is further believed in Malagasy traditional medicine to possess antiviral and anticancer properties [14, 15]. Preliminary studies of isolated *C. macrocarpa* derivatives identified capsicodendrin (CPCD) as a lead compound that was highly soluble and stable in aqueous environment, and capable of exerting cytostatic activity against a broad spectrum of cancer cell types including murine Leukemia cells (L1210/0), human T-lymphocyte cells (Molt4/C8 and CEM/0), HeLa and HT29 cells at sub-micromolar ranges [16].

Many chemotherapeutic agents possess anti-angiogenic properties as they produce cytotoxic or cytostatic effects by targeting cellular pathways that promote apoptosis or autophagy [17, 18]. The latter process has clear roles in various cellular and pathologic states, although its major function in angiogenesis is somewhat contentious [19–23]. Indeed, many studies have shown that autophagy inhibits angiogenic vasculature, whereas others have suggested its key role in neovessel formation. Several natural compounds capable of inducing autophagy-mediated inhibition of angiogenesis and cell death have already been reported, and in many instances, appear to target a broad range of cellular pathways including VEGF gene expression [17, 18, 22].

In the present study, we tested CPCD as a lead compound for potential anti-angiogenic activity and defined its mechanism of action. We report that CPCD has distinct autophagy-related angiostatic effects *in vitro* and *in vivo*.

## RESULTS

**Figure 1:**
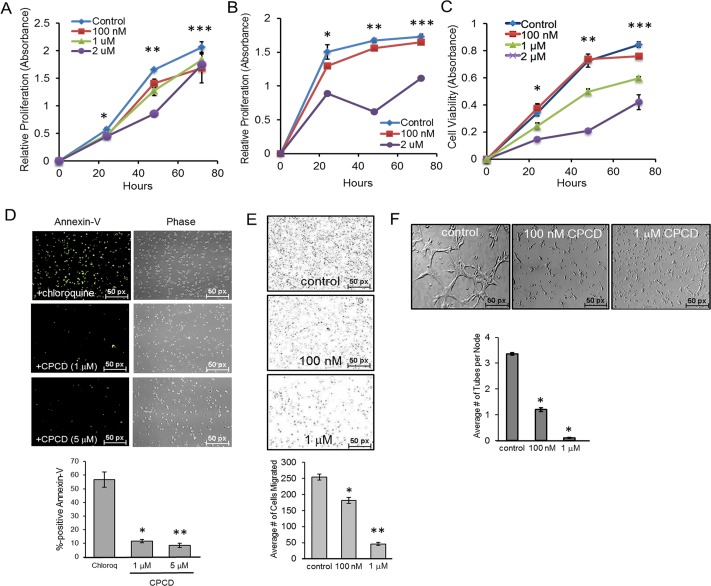
CPCD attenuates endothelial proliferation, migration, and capillary tube formation **A**. MTT growth assay of MEECs treated with CPCD (100 nM, 1 μM, 2 μM) for 24 h., 48 h., and 72 h. Presented data are an average readings from 6 independent experiments. ANOVA analysis of MTT assay readings at 24 h., 48 h., and 72 h. are as follows: *P* = 0.000135, *P* = 2.33 × 10^-14^, *P* = 0.003. Student's *T*-Test analyses are as follows: *P < 0.001 for control *vs* 100 nM, control *vs* 1 μM, control *vs* 2 μM, **P < 0.00005 for control *vs* 100 nM, control *vs* 1 μM, control *vs* 2 μM, ***P < 0.01 for control *vs* 100 nM, control *vs* 1 μM, and control *vs* 2 μM. **B**. MTT growth assay of HMECs treated with CPCD (100 nM, 1 μM, 2 μM) for 24 h., 48 h., and 72 h. Presented data are an average readings from 5 independent experiments. ANOVA analysis of MTT assay readings at 24 h., 48 h. 72 h. are as follows: *P* = 4.38 × 10^-20^, *P* = 2.039 × 10^-18^, *P* = 3.09 × 10^-12^. Student's *T*-Test analyses are as follows: *P < 0.05 for control *vs* 100 nM, **P < 0.0003 for control *vs* 100 nM, control *vs* 1 μM, and control *vs* 2 μM. **C**. Crystal violet growth assay of MEECs treated with CPCD (100 nM, 1 μM, 2 μM) for 24 h., 48 h., and 72 h. Presented data are average readings from 4 independent experiments. ANOVA analysis of crystal violet readings at 24 h., 48 h., and 72 h. are as follows: *P* = 2.048 × 10^-5^, *P* = 1.85 × 10^-8^, *P* = 5.51 × 10^-11^. Student's *T*-Test analyses are as follows: *P < 0.05 for control *vs* 100 nM, control *vs* 1 μM, and control *vs* 2 μM, **P < 9.1 × 10^-7^ for control *vs* 1 μM, and control *vs* 2 μM, ***P < 0.004 for control *vs* 100 nM, control *vs* 1 μM, and control *vs* 2 μM. **D**. Representative images of MEEC Annexin V staining upon chloroquine (1 μM) and CPCD treatment (1 μM, 5 μM) for 48 h. followed by quantification of the percentage of Annexin V positive cells per field. 10 random fields for each condition were analyzed from 4 independent experiments. ANOVA analysis are as follows: *P* = 4.08 × 10^-9^. Student's *T*-Test analyses are as follows: *P = 4.09 × 10^-6^, **P = 2.55 × 10^-6^. **E**. Representative images of MEEC transwells upon CPCD treatment (100 nM, 1 μM) for 16 h. followed by quantification of the average number of cells migrated. For each condition, 12 random fields were digitally imaged and counted from 4 independent experiments. ANOVA analysis are as follows:*P* = 4.3 × 10^-15^. Student's *T*-Test analyses are as follows: *P = 3.6 × 10^-5^, **P = 1.2 × 10^-12^. **F**. Representative images of three-dimensional Matrigel-induced capillary tubules for MEECs treated with CPCD (100 nM, 1 μM) for 6 h. followed by quantification of the average number of capillary tubes per node. For each condition, eight random fields were digitally imaged and analyzed from 4 independent experiments. ANOVA analyses are as follows: *P* = 2.02 × 10^-11^. Student's *T*-Test analyses are as follows: *P = 3.1 × 10^-7^, **P = 1.2 × 10^-9^.

Prior to investigating the potential angiostatic effects of CPCD, its chemical structure, molecular mass and stability were determined by NMR and mass spectrometry, and the overall purity of the isolated molecule from *C. macrocarpa* verified by total ion chromatography (see Supplementary material online, Figure S1). We first tested the effects of CPCD using the MTT growth assay in mouse embryonic endothelial cell line (MEEC) treated with or without increasing concentration of CPCD for up to 72 h. Here, a moderate to significant growth-inhibition was observed at concentrations ranging from 100 nM to 2 µM at 48 h (Figure 1A), a result that was recapitulated in a parallel study involving human microvascular endothelial cell 1 (HMEC1) (Figure 1B). Similar results were obtained using crystal violet colorimetric assay as a read-out of cell proliferation, and found a largely concentration-dependent growth-inhibition was observed over the course of 72 h upon drug treatment (Figure 1C). To test whether apoptosis contributed to the overall growth inhibition, annexin-V staining was performed in MEECs and isolated primary mouse aortic endothelial cells (MAECs). Compared to control chloroquine treatment, CPCD did not promote apoptosis even at higher concentrations up to 72 h (Figure 1D and graph, see Supplementary material online, Figure S2), suggesting that CPCD exerts cytostatic effects on endothelial cells. Next, we assessed the effects of CPCD on cell motility using the Boyden transwell chamber system. Relative to control, CPCD inhibited endothelial migration in a concentration-dependent manner (Figure 1E and graph), a finding that was consistent with the dose-dependent inhibition of capillary tube formation in three-dimensional matrigel assay using MEECs and MAECs (Figure 1F and graph, see Supplementary material online, Figure S3A). Taken together, these results indicated that CPCD acts as a potent angiostatic compound *in vitro*.

**Figure 2:**
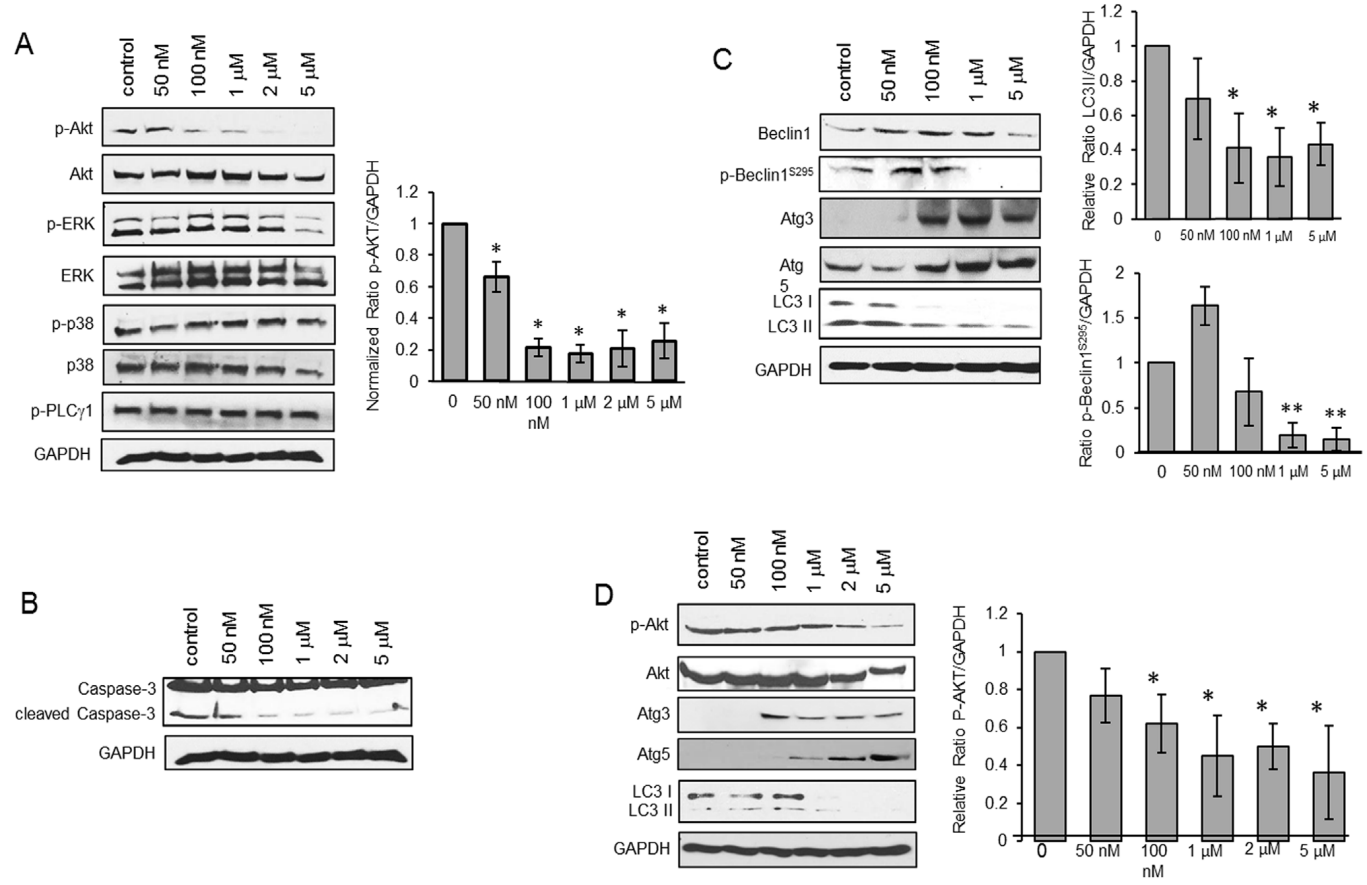
CPCD induces autophagy by impairing Akt activation **A**. Western analysis of p-Akt protein levels in MEECs treated with CPCD (50 nM, 100 nM, 1 μM, 2 μM, 5 μM) for 2 h. followed by densitometry analysis of p-Akt levels normalized to GAPDH from three independent experiments. Student's *T*-Test analyses are as follows: *P < 0.04. **B**. Western analysis of caspase-3 cleavage in MEECs treated with CPCD (50 nM, 100 nM, 1 μM, 2 μM, 5 μM) for 2 h. **C**. Western analysis of autophagic markers in MEECs treated with CPCD (50 nM, 100 nM, 1 μM, 2 μM, 5 μM) for 2 h. followed by densitometry analysis of LC3 II levels and p-Beclin1^S295^ levels normalized to GAPDH from three independent experiments. Student's *T*-Test analyses are as follows: *P < 0.04, **P < 0.005. **D**. Western analysis of autophagic markers in MEECs and HMECs treated with CPCD (50 nM, 100 nM, 1 μM, 2 μM, 5 μM) for 2 h. followed by densitometry analysis of p-Akt levels normalized to GAPDH from three independent experiments. Student's *T*-Test analyses are as follows: *P < 0.05.

**Figure 3:**
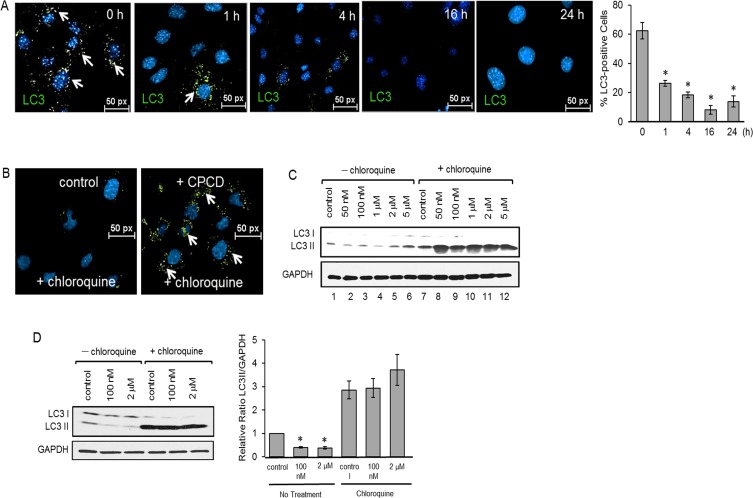
CPCD rapidly enhances LC3 I/II degradation **A**. Representative images of MEECs stained for LC3 I/II after treatment with CPCD (100 nM) for 1 h., 4 h., 16 h., and 24 h. followed by quantification of percentage of LC3 I/II positive cells. For each condition, 13 random fields were digitally imaged and analyzed. ANOVA analyses are as follows: *P* = 2.77 × 10^-9^. Student's *T*-Test analyses are as follows: *P < 0.000144. **B**. Representative images of MEECs treated with CPCD (100nM) and chloroquine (1 μM) for 2 h. and stained for LC3 I/II. **C**. Western analysis of LC3 cleavage in MEECs cotreated with CPCD (50 nM, 100 nM, 1 μM, 2 μM, 5 μM) and chloroquine (1 μM) for 2 h. D) Western analysis of LC3 cleavage in MAECs cotreated with CPCD (100 nM, 2 μM) and chloroquine (1 μM) for 2 h. followed by densitometry analysis of LC3 II levels normalized to GAPDH from three independent experiments. ANOVA analyses are as follows: *P* < 0.0001. Student's *T*-Test analyses are as follows: *P < 0.0003.

To define the underlying mechanism, we screened for angiogenic pathways affected by CPCD including ERK, p38, PLCγ, among others (Figure 2). Despite its potent cytostatic effects, CPCD had minimal impact on mitogenic signaling such as ERK and p38 (Figure 2A). Instead, there was a notable concentration-dependent AKT inactivation (Figure 2A and graph). Despite its key role in cell survival, AKT signaling attenuation had little impact on caspase-3 cleavage even at up to 5 µM (Figure 2B), and hence agreed with our annexin-V results.

Aside from cell survival, AKT mediates a number of other cellular processes including migration, metabolism and autophagy. Given that AKT is a suppressor of autophagy in most systems, we examined whether the CPCD-dependent AKT inactivation promotes autophagy by measuring the relative levels of autophagic markers including beclin1, Atg3, Atg5 and microtubule-associated protein light chain 3 (LC3) (Figure 2C). A 2 h treatment with increasing concentrations of CPCD in both MEECs and HMEC1 resulted in elevated levels of beclin1, Atg3 and Atg5 expression (Figure 2C and 2D). Moreover, there was a corresponding decrease in inhibitory beclin1 phosphorylation by AKT, strongly suggesting that CPCD is an inducer of autophagy (Figure 2C). Surprisingly, the final step of autophagy involving LC3-I conversion to LC3-II significantly decreased in a dose-dependent manner in our biochemical analysis and time-course study as monitored by immunofluorescence staining of LC3-positive autophagosomes (Figure 2C; fourth panel and Figure 3A and graph). As LC3-II itself is degraded during autophagy in the lysosome, we speculated that CPCD markedly accelerates LC3-II degradation. To test this, we pre-treated MEECs with a lysosomal inhibitor chloroquine in the presence or absence of CPCD and found a striking increase in LC3-II autophagasome vesicles (Figure 3B; arrows). Similar results were obtained in our biochemical analysis wherein the basal level of LC3-II rose only modestly upon lysosomal inhibition while CPCD treatment drastically raised LC3-II levels under the same condition in MEECs and primary MAECs (Figure 3C and 3D, and see Supplementary material online, Figure S3). Collectively, these results indicated that CPCD induces endothelial autophagy by targeting AKT activation.

**Figure 4:**
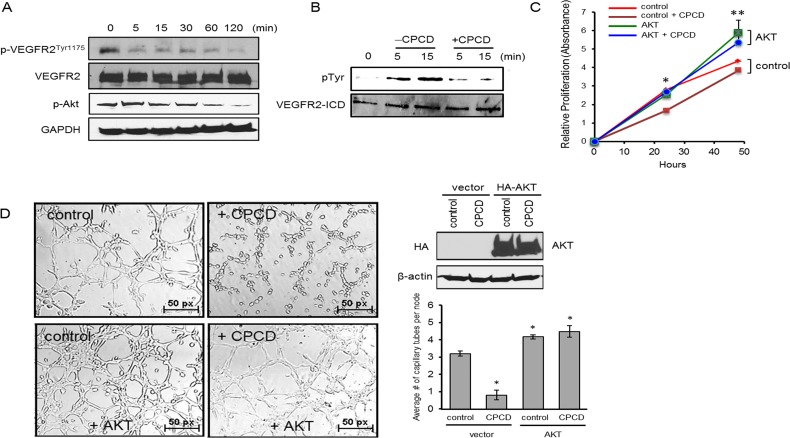
CPCD impairs Akt activation by inhibiting VEGFR2^Tyr1175^ phosphorylation **A**. Western analysis of p-VEGFR2^Tyr1175^ levels and p-Akt levels in MEECs treated with CPCD (100 nM) for the indicated time (5 min., 15 min., 30 min., 60 min., 120 min.) **B**. Western analysis of VEGFR2 intracellular tyrosine phosphorylation upon 5 µM CPCD treatment (5 min., 15 min.) **C**. Crystal violet growth assay of MEECs overexpressing Akt and treated with CPCD (2 μM) for 24 h. and 48 h. Presented data are average readings from 4 independent experiments. ANOVA analyses are as follows: *P = 7.4 × 10^-12^, **P = 0.005. Student's *T*-Test analyses are as follows: *P = 2.0 × 10^-9^ for control *vs* control + CPCD, **P = 0.01 for control *vs* control + CPCD. **D**. Representative images of three-dimensional Matrigel-induced capillary tubules for MEECs overexpressing Akt after CPCD treatment (100 nM) for 6 h. followed by quantification of the average number of capillary tubes per node and western analysis of HA-Akt overexpression. 10 random fields were digitally imaged and analyzed for each condition from 4 independent experiments. ANOVA analyses are as follows: *P = 5.38 × 10^-10^. Student's *T*-Test analyses are as follows: *P = 2.49 × 10^-6^, **P < 0.0005.

We next sought to determine the mechanism by which CPCD blocks AKT activation and downstream signaling. Given its potent angiostatic effects, we tested whether this compound targets vascular endothelial growth factor receptor 2 (VEGFR2), a major upstream activator of AKT in the endothelial system. Indeed, a time-course experiment upon drug treatment followed by a biochemical analysis of receptor autophosphorylation revealed VEGFR2 inactivation within minutes, particularly at Tyr1175, which mediates the recruitment and activation of the PI3-kinase and subsequent AKT signaling (Figure 4A) [24]. To determine whether CPCD directly targets VEGFR2, the intracellular segment comprising the receptor kinase and C-terminal autophosphorylation domain was purified in baculovirus/insect cell system and subjected to an *in vitro* kinase assay in the presence or absence of CPCD pretreatment. Upon initiating the kinase reaction with ATP, there was rapid kinase activation as evidenced by tyrosine autophosphorylation at 5 min, and continued to rise at 15 min (Figure 4B, see Supplementary material online, Figure S4A). In contrast, pre-incubating the purified protein with CPCD prior to ATP addition prevented receptor autophosphorylation, indicating that the small molecule acts as an inhibitor of VEGFR2 kinase (Figure 4B). Since VEGFR2 kinase inactivation helps explain how CPCD attenuates AKT signaling, we next evaluated the role of AKT-targeting in endothelial proliferation (Figure 4C). Here, CPCD treatment resulted in reduced proliferation in control MEECs, whereas AKT overexpression enhanced basal proliferation and counteracted the growth-inhibitory effects of CPCD (Figure 4C). Moreover, the matrigel capillary tube assay yielded a similar pattern in which, relative to control, AKT overexpressing MEECs resisted the overall angiostatic effects of CPCD (Figure 4D and graph). Taken together, these results strongly supported AKT as a major inhibitory target of CPCD during angiogenesis.

**Figure 5:**
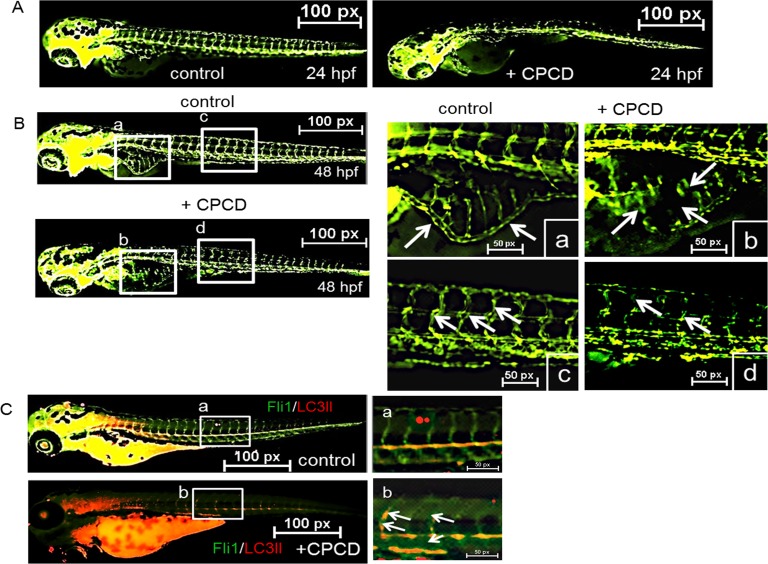
CPCD negatively regulates sprouting angiogenesis *in vivo* **A**. Representative images ofTg(fli1:eGFP) embryos treated with CPCD (2 μM) for 24 h. **B**. CPCD (2 μM) treated embryos displayed impaired sprouting of ISVs, an absence of the DLAV, and a reduction of the SIV. 30 embryos were analyzed to determine the angiogstatic effects of CPCD. **C**. Representative images of zebrafish embryos stained for LC3II (red).

Lastly, we evaluated the angiostatic effects *in vivo* by monitoring the effects of CPCD on Tg(*fli1:eGFP*) zebrafish during embryonic development (Figure 5) [27]. Here, fish embryos were grown in petri dish containing 2 µM of CPCD. Compared to control, CPCD treatment exerted potent anti-angiogenic effects within the first 24 hours post-fertilization (hpf), as indicated by reduced vascular sprouting of intersegmental vessels (ISV) (Figure 5A arrows). Upon 48 hpf, significant angiogenic defects were evident including abnormal subintestinal vessel (SIV) formation and markedly diminished SIV sprouts (Figure 5B; a-d arrows). Importantly, while CPCD treatment significantly diminished the overall sprouting of the ISVs relative to control, more LC3-II vesicles were present along the defective ISVs (Figure 5C arrows), supporting our *in vitro* data that CPCD inhibits angiogenesis by disrupting the regulation of endothelial autophagy.

## DISCUSSION

Our work defines novel cytostatic properties of a natural compound isolated from *C. macrocarpa.* Aside from its use as traditional medicine for numerous ailments, CPCD demonstrates selective inhibition of the VEGFR2 kinase and downstream AKT activation. Our data further supports enhanced autophagy as the major mechanism by which it inhibits angiogenesis, although other ATK-related cellular functions such as metabolism still remain to be explored.

Considerable progress has been made over the years with targeted anti-cancer and anti-angiogenic therapies, and natural compounds have undoubtedly played an integral role in drug-discovery efforts. Indeed, there is an ever-growing inventory of small molecule inhibitors derived from natural products, many of which are currently under preclinical or clinical evaluations [9]. However, achieving high potency and selectivity on the desired target continue to be the principal challenge even among many promising drugs in the pipeline. Along these lines, our study demonstrates CPCD as a promising lead compound given its *in vitro* efficacy at nanomolar concentrations. Still, despite its potent angiostatic effects, CPCD showed signs of potential off-target cardiovascular effects *in vivo*, as we observed a dose-dependent decrease in the heart rate of developing zebrafish embryos at 72 hpf (see Supplementary material online, Figure S4B). Although no gross changes were evident in terms of the overall size of the embryos (~1-2 mm diameter) or the heart structure compared to control (data not shown), additional studies are required to fully characterize the cardiovascular effects of CPCD in developing *versus* adult vertebrate systems.

While many natural compounds previously have shown inhibitory properties of VEGF signaling, the precise mechanisms are often unclear as they broadly target multiple cellular pathways including VEGF, VEGR2, AKT, MAPKs, hypoxia-inducible factors (HIFs) and many others [11]. Hence, it is noteworthy that CPCD appears to be more selective in terms of directly targeting the VEGFR2 kinase and attenuating AKT activity at nanomolar concentrations while ERK and other mitogenic signaling remain unperturbed (Figure 2 and 4A, 4B). Although it is not clear whether CPCD also inhibits other tyrosine kinases, we found that it did not inhibit the activation of at least one other receptor tyrosine kinase, the insulin receptor, in endothelial cells under similar conditions (data not shown). Understanding the molecular mechanism by which CPCD inhibits VEGFR2 autophosphorylation would require direct structure-activity relationship studies involving *in silico* docking and biophysical characterization.

Autophagy is a dynamic process of subcellular degradation that mediates cell survival under nutrient-deprivation, hypoxia and many other stress-related conditions. The precise role of autophagy in angiogenesis is contentious with many conflicting reports indicating either anti-angiogenic or pro-angiogenic properties. In our system, CPCD proved to be a strong autophagy inducer capable of enhancing the major autophagy-related components including beclin1, Atg3 and 5, as well as producing high-rate turnover of LC3-II degradation. More importantly, our *in vivo* findings establish a link between the angiostatic effects of CPCD and increased autophagy within defective sprouting vessels. Despite the controversy, basal autophagy is presumably required for angiogenesis, as it clears damaged organelles, long-lived proteins and has other protective functions during a time of high metabolic demand. In contrast, excessive self-digestion dramatically increases the formation of reactive oxygen species (ROS) that induces cytotoxicity in many cell types including the endothelial system. Our data suggests that CPCD tips this critical balance towards excessive autophagy that impairs critical endothelial functions during angiogenesis.

Taken together, our work defines the potent angiostatic role of CPCD, a promising lead compound that serves as a basis for the development of new derivatives and combination therapy studies in preclinical settings.

## MATERIALS AND METHODS

### Cell culture, plasmids, transfections, and antibodies

All mouse and zebrafish studies were performed in accordance with the guidelines provided by the Institutional Animal Care and Use Committee (IACUC) at The Ohio State University and the NIH guidelines (Guide for the care and use of laboratory animals). All established endothelial cell lines used were of comparable passage number of 10-20. Mouse embryonic endothelial cell line (MEECs) was derived from mice at E9 and cultured as previously described [23, 25]. Briefly, C57BL/6 mice purchased from Jackson Laboratories (Maine). Mice were euthanized according to the NIH and The Ohio State University approved guidelines, involving intraperitoneal injection of 300 mg/kg body weight sodium pentobarbital. MEECs were maintained in MCDB-131 medium (Invitrogen) supplemented with 2 mM L-glutamine, 1 mM sodium pyruvate, 15% fetal bovine serum, 100 µg/ml heparin, 25 µg/ml endothelial cell growth supplement. Human microvascular endothelial cell line (HMEC-1) was purchased through ATCC and maintained in MCDB-131 medium supplemented with 10% fetal bovine serum, 1 µg/ml hydrocortisone (Sigma), 10 ng/ml epidermal growth factor (Sigma), and 2 mM L-glutamine. Primary mouse aortic endothelial cells (MAECs) were isolated and cultured as we previously described and used at low passage numbers ( < 10) [26]. Briefly, C57BL/6 mice purchased from Jackson Laboratories. Mice were euthanized according to the NIH and The Ohio State University approved guidelines, involving intraperitoneal injection of 300 mg/kg body weight sodium pentobarbital. MAECs were maintained in 40% DMEM, 40% HAM, 20% fetal bovine serum, 30 mg/ml endothelial cell growth supplement, 100 µg/ml heparin. Transfections were achieved by using Lipofectamine 2000 as described according to manufacturer protocol (Invitrogen). HA-Akt construct was a generous gift from Dr. Frederick W. Quelle, University of Iowa. Basement matrigel matrix was obtained from BD Bioscience. The lysosome inhibitor chloroquine, β-actin, and anti-HA antibodies were purchased from Sigma-Aldrich. P-Beclin1^S295^ was purchased from Abcam. Anti-phosphotyrosine antibody, PY20, was purchased from Millipore. The following antibodies were all purchased from Cell Signaling: phospho-Akt (no. 13038), total Akt (no. 4685), phospho-ERK (no. 9101), total ERK (no. 9102), phospho-p38 (no. 4511), total p38 (no. 8690), phospho-PLCγ1 (no. 14008), GAPDH (no. 5174), Beclin-1 (no. 3495), Atg3 (no. 3415), Atg5 (no. 12994), LC3 A/B (no. 4108), Cleaved Caspase-3 (no. 9664), p-VEGFR2^Tyr1175^ (no. 2478), total VEGFR2 (no. 9698), Annexin V-FITC early apoptosis detection kit (no. 6592).

### Immunofluorescence

MEECs and MAECs were grown overnight on coverslips and treated with CPCD (100 nM) alone or CPCD (100 nM) in the presence of chloroquine (1μM) for the indicated duration. Following treatment, cells were fixed with 4% paraformaldehyde. Cells were permeabilized in 0.1% Triton X-100 in PBS for 3-10 min, then blocked with 5% bovine serum albumin in PBS containing 0.05% Triton X-100 for 20 min. All primary Abs were incubated at room temperature for 1 h unless noted otherwise. LC3 A/B Ab was used to detect autophagosome clusters. Following primary antibody incubation, cells were incubated with appropriate flurophore conjugated secondary antibodies (Alexa-Fluor) at room temperature for 30 min. Cells were co-stained with DAPI (Sigma) immediately before immunofluorescence microscopy analyses (Nikon Eclipse Ti). Autophagy levels were quantified by counting the number of autophagosome-positive cells.

### Annexin V-FITC staining

MEECs and MAECs were grown overnight 10 cm plates and treated with CPCD (1 μM, 5 μM) for 48 h. and 72 h. Following treatment, cells were trypsinized, pelleted, and resuspsended with 500 μl of 1X Annexin V Binding Buffer. Following Annexin V Binding Buffer, 1 μl of Annexin V-FITC conjugated antibody and 12.5 μl of propidium iodide were added. Cells were incubated in the dark on ice for 10 mins and then analyzed by immunofluorescence microscopy and ImageJ.

### CPCD isolation and molecular characterization

Powdered C. fragrans was extracted with methanol and then purified by column chromatography on silica gel (solvent: hexane-ethyl acetate gradient). Purification of fractions were characterized by ^1^H and ^13^C NMR on Varian Unity 600 NMR using CDCl_3_ as a solvent. Mass spectra were recorded on JEOL JMS AX-500 spectrometer. Column chromatography was carried out on Sephadex LH-20 (Amersham) and silica gel. Preparative HPLC was performed using a reversed-phase column.

### Endothelial tube formation

MEECs or MEECs transiently overexpressing the wild type mouse Akt were plated on a 24-well plate coated with 200 μl of matrigel basement matrix (Corning) at 140,000 cells/well in the presence of CPCD at the indicated doses. 1 h following plating, growth medium was removed and 200 μl of matrigel was added. 30 min following the addition of the matrigel basement matrix, 300 μl of growth medium containing the indicated doses of CPCD was added. Endothelial tubes were digitally imaged and quantified by counting the number of branches per node.

### Transwell migration assays

MEECs were seeded in the upper chamber of a transwell filter in complete growth media containing CPCD at various doses, coated both at the top and bottom with gelatin. Cells were allowed to migrate for 12 h toward the lower chamber. Cells that migrated to the bottom surface of the filter were fixed, stained, and then digitally imaged and counted.

### Crystal violet and MTT growth assay

MEEC control and cells transiently overexpressing Akt were plated at 15,000 in 12-well plates and treated with different doses of CPCD. Cells were fixed at different time points (4% paraformaldehyde in PBS for 15 min). Following fixation, cells were washed with 1× water and stained with 0.1% crystal violet for 20 min. Cells were washed 3× with water and allowed to air dry for 30 min. Cells were destained using crystal violet de-staining solution (10% acetic acid, 50% methanol, 40% H_2_0) for 20 min, and the optical density was read at 590 nm in a microplate reader. Similarly, the MTT assay MEECs were plated at 15,000 in 12-well plates and treated with different doses of CPCD for 24, 48 and 72 h. Following incubation, the media was aspirated and replaced with 500 µl of MTT (5 mg/mL) for 3h at 37 C. MTT was aspirated and dissolving buffer (4mM HCl, 0.1% NP40 in isopropanol) was added and the absorbance was measured at 590 nm in a microplate reader

### Cloning and generation of recombinant baculovirus

Gene encoding the intracellular domain (ICD) of VEGFR2 (790-1356 amino acids) was cloned into pFastBac1 vector (Life Technologies) between the NotI and XhoI restriction sites upon PCR amplification using a forward hexahistidine-encoding primer(5-GCGCGGCCGCGCCATGGATGCACCACCACCACCACCACAAGCGGGCCAATGAGGGG-3) and a reverse primer (5-CCGCTCGAGTTAAACAGG AGGAGAGCT-3). pFastBac1-VEGFR2-ICD plasmid was transformed into DH10Bac competent cells for production of bacmid DNA. Isolated bacmid DNA was transfected into Sf21 insect cells using Cellfectin II reagent (Invitrogen) according to protocols provided by manufacturer. Purified recombinant viruses were amplified with three rounds of infection in Sf21 cells grown at 27 C using a multiplicity of infection (MOI) of 0.1. Viral supernatants were harvested 48-72 h post infection.

### Purification of recombinant protein

Sf21 cells were infected with the baculovirus at MOI of 2. After 48 h post-infection, cells were harvested by centrifugation at 1000 g for 3 min, gently washed with resuspension buffer (20 mM HEPES pH 7.4, 0.5 M NaCl, 250 mM sucrose, protease inhibitors [5 µg/ml aprotinin, 5 µg/ml leupeptin, 2 µM pepstatin A, 1mM PMSF]). Cells were resuspended with sucrose-free resuspension buffer and then lysed by sonication on ice. After sonication, cell lysate was centrifuged at 11,000 rpm for 20 min prior to being supplemented with 0.05% Triton X-100 and then loaded onto a cobalt resin column (Thermo Scientific) pre-equilibrited with wash buffer (20 mM HEPES pH 7.4, 0.5 M NaCl, 15 mM imidazole, 0.05% TX-100). The column was washed with at least 20 column-volumes of wash buffer before elution with elution buffer (20mM HEPES pH 7.4, 0.5 M NaCl, 150 mM imidazole, 0.05% TX-100) and 0.5 ml fractions were collected and checked for protein concentration prior to being pooled and dialyzed extensively against 20mM HEPES (pH 7.4), 100 mM NaCl at 4 C.

### *In vitro* protein tyrosine kinase assay

Kinase assays were typically performed at 25 C by pre-incubating 0.5 µM of purified VEGFR2-ICD protein with 1 mM MnCl_2_ for 5 min in kinase assay buffer (20 mM HEPES at pH 7.4, 150 mM NaCl) and then 100 µM ATP was added. The reactions were stopped after the noted period (5 and 15 min) with SDS-PAGE sample buffer. Protein reactions were resolved by SDS-PAGE and immunoblotted with VEGFR2 (Cell signaling) and anti-phosphotyrosine (PY20, Millipore).

### Zebrafish vascular sprouting during embryonic development

Tg(fli1:eGFP) zebrafish embryos were obtained from Dr. Christine Beattie under the auspices of the NINDS P30 Zebrafish Genetics Core at the Ohio State University and grown in 10 cm petri dish filled fish water containing 2 μM CPCD. 24 and 48 hours post fertilization, Fli1:EGFP zebrafish embryos were treated with tricaine (160 μg/mL) and digitally analyzed using immunofluorescence microscopy (Nikon Eclipse Ti). For immunostaining, 48 h. embryos were fixed with 4% paraformaldehyde for 2 h. and placed in 100% methanol for 2 h at -20°C. Embryos were then blocked using blocking buffer 1x PBST, 10% heat-inactivated fetal bovine serum, 2% bovine serum albumin) for 1 h. at room temperature. Following blocking, embryos were incubated with LC3 A/B primary antibody (Cell Signaling) at 4°C overnight. Following primary antibody incubation, embryos were incubated with anti-rabbit flurophore conjugated secondary antibody for 4 h. at room temperature. Relative LC3 A/B levels and localization were analyzed using immunofluorescence microscopy (Nikon Eclipse Ti).

### Zebrafish embryo heart rate analysis

Transgenic zebrafish were grown in 10 cm petri dish filled fish water containing 1 μM and 5 μM CPCD. At 24, 48 and 72 h hpf zebrafish hearts were visualized using immunofluorescence microscopy and heart rates were determined by counting the number of heartbeats per minute.

### Statistics

Statistical analysis was performed using both student *t*-test and one-way anova. Data are presented as mean ± SEM. Student *t*-test was performed to determine statistical significance for densitometry analysis of western blots. Statistical significance for all other experiments was determined using one-way ANOVA. *P* values of < 0.05 were considered as significant.

## SUPPLEMENTARY MATERIALS FIGURES


