# Supplementary material for: Angiostatic actions of capsicodendrin through selective inhibition of VEGFR2-mediated AKT signaling and disregulated autophagy
Source: Oncotarget. 2016 May 11;8(8):12675–85. doi: 10.18632/oncotarget.9307 (PMC5355044; doi:10.18632/oncotarget.9307)
Supplement: Supplementary file 1 [file oncotarget-08-12675-s001.pdf]

Angiostatic actions of capsicodendrin through selective inhibition of VEGFR2-mediated AKT signaling and disregulated autophagy

Supplementary Material

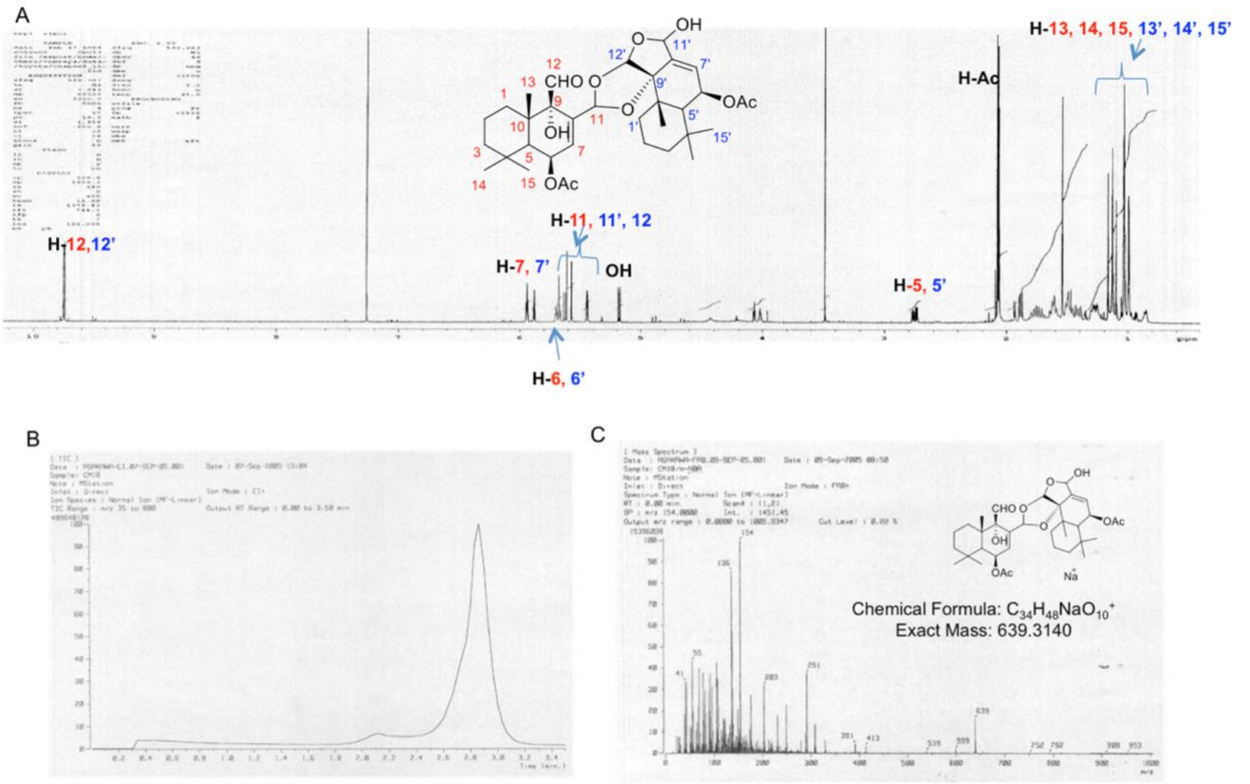

Supplementary Figure 1: A) ^{1}H-NMR Spectrum of Capsicodendrin (600 MHz, $CDCl_3$). B) Total Ion Chromatography (TIC) of Capsicodendrin (CM18). Ion mode: EI. C) Positive FAB-MS Spectrum of Capsicodendrin (CM18).

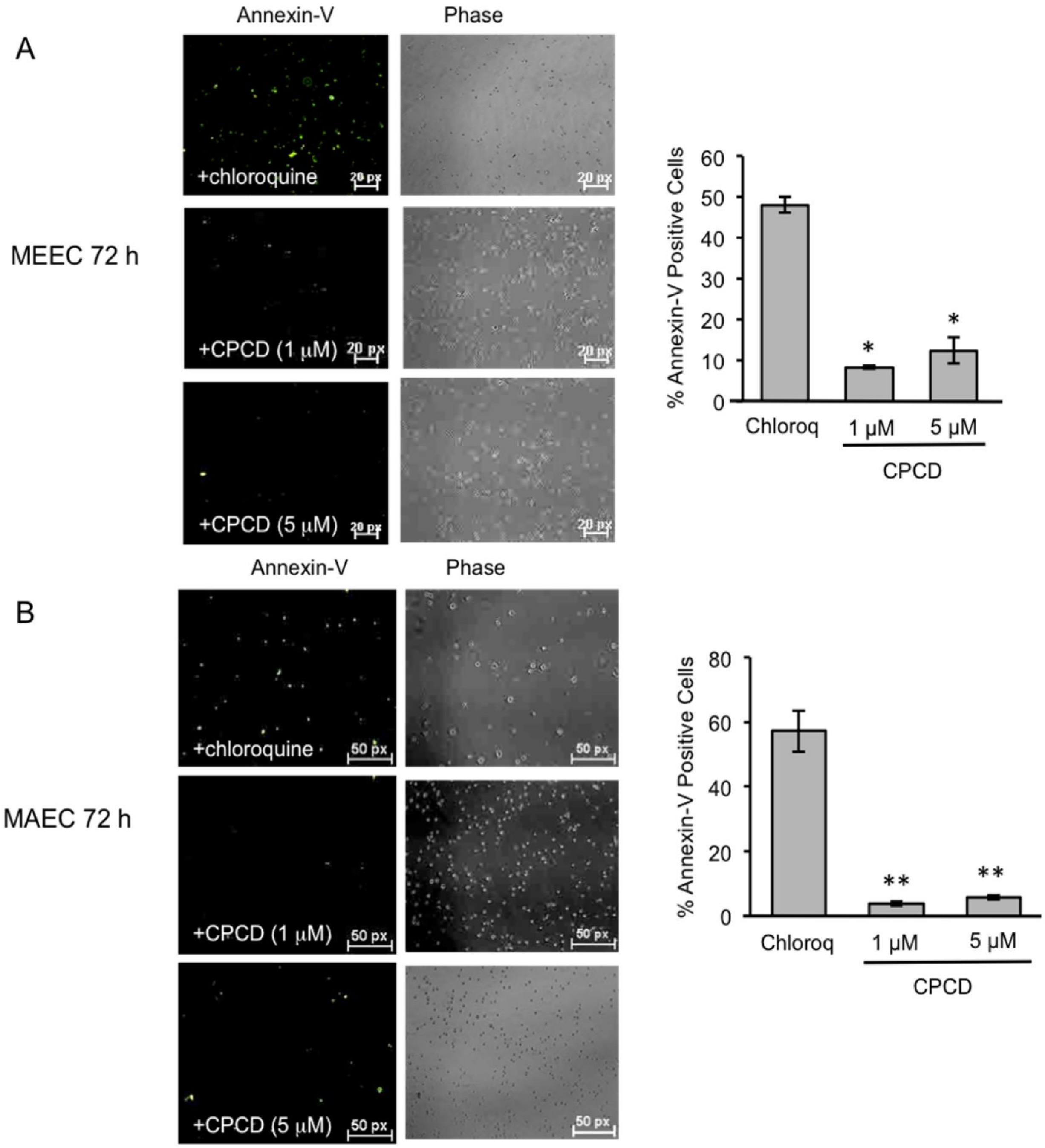

Supplementary Figure 2: CPCD treatment does not induce cell apoptosis. A). Representative images of Annexin-V staining in MEECs and MAECs treated with chloroquine (1 μM) and CPCD (1 μM, 5 μM) for 72 h. followed by quantification of percentage of Annexin-V positive cells per field. For each condition, 13 random fields were digitally imaged and analyzed. ANOVA analyses for Annexin-V staining in MEECs and MAECs 72 h. are as follows: $P=1.68 \times 10^{-10}$, $P=5.49 \times 10^{-15}$. Student's T-Test analyses are as follows: $^{*}P<1.89 \times 10^{-9}$, $^{**}P<6.82 \times 10^{-11}$.

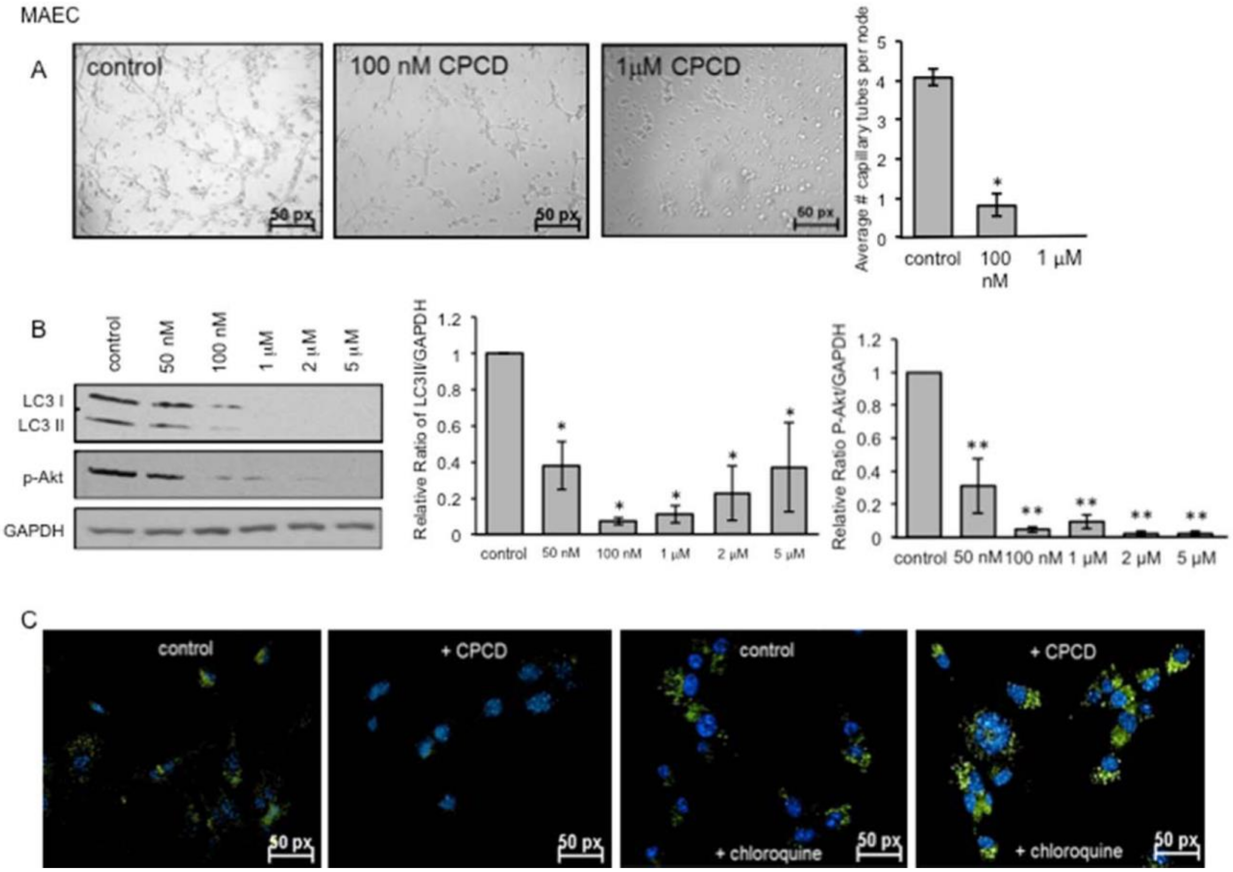

Supplementary Figure 3: CPCD induces autophagy by impairing Akt activation in primary cells. A) Representative images of three-dimensional Matrigel-induced capillary tubules for MAECs after CPCD treatment (100 nM) for 8 h. followed by quantification of the average number of capillary tubes per node. ANOVA analyses are as follow $P=3.96 \times 10^{-10}$. Student's T-Test analyses are as follows: $*P<1.58 \times 10^{-6}$. B) Western analysis of LC3 cleavage and p-Akt levels in MAECs treated with CPCD (50 nM, 100 nM, 1 μM, 2 μM, 5 μM) for 2 h. followed by densitometry analysis of LC3 II levels and p-Akt levels normalized to GAPDH from three independent experiments. Student's T-Test analyses are as follows: *P<0.05, **P<0.02 C) Representative images of MAECs treated with CPCD (100nM) and chloroquine (1 μM) for 2 h. and stained for LC3 I/II. For each condition, 15 random fields were digitally imaged and analyzed.

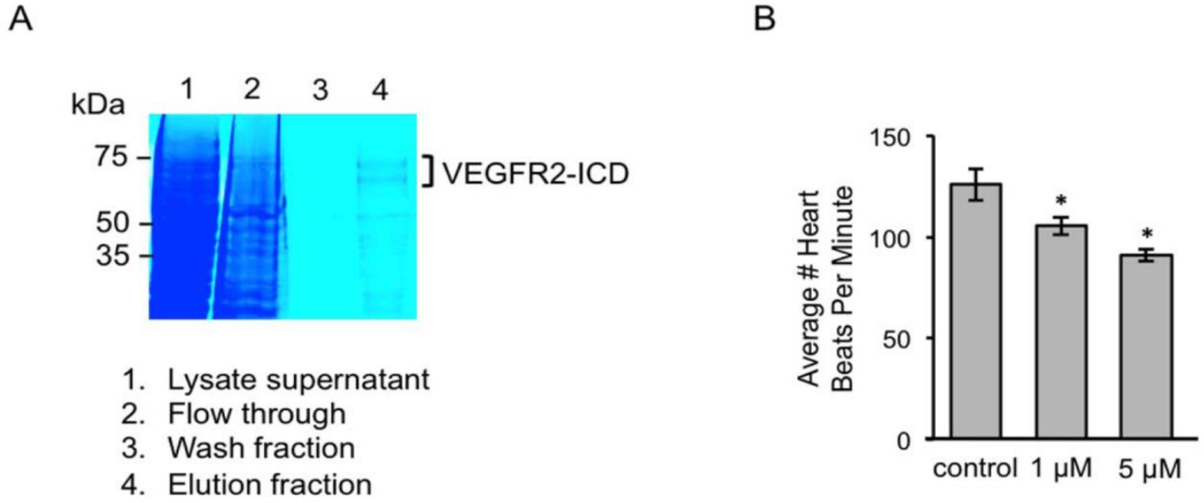

Supplementary Figure 4: Quantification of Tg(fli1:eGFP) zebrafish heart rates upon treatment with CPCD (1 μM, 5 μM) for 72 h. post fertilization. Eight zebrafish embryos were analyzed for each condition. ANOVA analyses are as follows: $P=0.001$. Student’s T-Test analyses are as follows: $^{*}P<0.05$.
